# Bidirectional causal link between inflammatory bowel disease and celiac disease: A two-sample mendelian randomization analysis

**DOI:** 10.3389/fgene.2022.993492

**Published:** 2022-09-20

**Authors:** Gu A, Caixia Sun, Yuezhan Shan, Husile Husile, Haihua Bai

**Affiliations:** ^1^ Department of Gastrointestinal Colorectal and Anal Surgery, China-Japan Union Hospital of Jilin University, Changchun, China; ^2^ Affiliated Hospital of Inner Mongolia University for the Nationalities, Tongliao, China; ^3^ Baotou Medical College, Inner Mongolia University of Science and Technology, Baotou, China

**Keywords:** inflammatory bowel disease, ulcerative colitis, Crohn ‘s disease, Mendelian randomization, celiac

## Abstract

**Background:** Observational research has shown a correlation between inflammatory bowel disease (IBD) [comprising ulcerative colitis (UC) and Crohn’s disease (CD)] and celiac disease. However, the relationship between these two diseases remains uncertain.

**Methods:** We utilized two-sample Mendelian randomization (MR) to estimate the bidirectional causal relationships between IBD and celiac disease. This study utilized data on single nucleotide polymorphisms (SNPs) from genome-wide association studies (GWASs). Heterogeneity, pleiotropy, and sensitivity analyses were also performed to evaluate the MR results.

**Results:** There was a significant causal relationship between IBD and CD and celiac disease (e.g., IBD and celiac disease, inverse variance weighting (IVW) odds ratio (OR) = 1.0828, 95% CI = 1.0258–1.1428, *p* = 0.0039; CD and celiac disease, IVW OR = 1.0807, 95% CI = 1.0227–1.1420, *p* = 0.0058). However, in the reverse direction, we found only suggestive positive causality between celiac disease and CD (e.g., IVW OR = 1.0366, 95% CI = 1.0031–1.0711, *p* = 0.0319). No evidence of heterogeneity between genetic variants was found (e.g., IBD vs. celiac disease, MR-Egger Q = 47.4391, *p* = 0.6159). Horizontal pleiotropy hardly influenced causality (e.g., IBD vs. celiac disease, MR-Egger test: *p* = 0.4340). Leave-one-out analysis showed that individual SNPs did not influence the general results.

**Conclusion:** Our MR analysis revealed a positive causal link between IBD and celiac disease in the European population. In addition, several recommendations for disease prevention and clinical management have been discussed.

## Introduction

Celiac disease is an immune disease that affects approximately 1% of the world’s population and is characterized by intolerance to wheat gluten, which causes intestinal mucosal lesions (Lebwohl et al., 2018). Although the incidence of celiac disease has dramatically increased in the 21st century (King et al., 2020), it is frequently misdiagnosed because of its multifaceted clinical presentation (Singh et al., 2018). Like various autoimmune illnesses, genetics is the most prominent factor contributing to celiac disease (Kuja-Halkola et al., 2016). Gluten intake is an environmental factor that directly affects celiac disease. Inflammatory bowel disease (IBD) is a chronic inflammatory bowel disease comprising ulcerative colitis (UC) and Crohn’s disease (CD).

It has been previously reported that there is a link between IBD and celiac disease. According to a systematic evaluation, patients with CD are >11 times more likely to develop IBD than the general population (Shah et al., 2019). It has been reported that CD is more related to celiac disease than UC, and IBD patients treated with drugs had a low association with celiac disease (Alkhayyat et al., 2022). According to a previous study, UC and celiac disease share the same genetic background (Glas et al., 2009). Half a century has passed since Salam and Truelove first described the relationship between IBD and celiac disease; however, the causal relationships between the two illnesses are obscure (Jankey and Price, 1969).

Exploring the causal link between IBD and celiac disease contributes to a better understanding of its pathogenesis and, as a result, to improve treatment guidance. Randomized controlled trials (RCTs) are generally challenging, expensive, and complex to design and perform. Therefore, we adopted Mendelian randomization (MR) to investigate the potential causal link. The causal link between the exposure factor and outcome is studied by introducing an intermediary variable known as the instrumental variable (IV). Three assumptions should be fulfilled for the IV: (1): risk factors and genetic variation are related (2), genetic variation does not depend on confounding factors, and (3) genetic variation affects outcomes through risk factors only. The genetic variations used in MR are currently being utilized in large-scale genome-wide association studies (GWAS). We identified single nucleotide polymorphisms (SNPs) that were highly linked to IBD (UC and CD) as IVs. We used the effect of the IV on exposure (IBD, UC, and CD) and outcome (celiac disease) from two independent samples. A two-sample MR was used to analyze the effect of IBD on celiac disease. In addition, reverse MR was used to investigate the bidirectional causal link between IBD and celiac disease.

## Materials and methods

### Data source

A core step of our study was to screen SNPs as exposure and outcome IVs related to IBD (UC and CD) and celiac disease based on GWAS results and the literature. SNPs related to IBD were acquired from the IBD Genetics Consortium GWAS study (Liu et al., 2015). IBD is diagnosed by GWAS through radiographic, endoscopic, and pathological studies. The celiac GWAS provided pooled data on the influence of IBD-related SNPs on celiac disease (European population, comprising 12,041 cases and 12,228 population controls) (Trynka et al., 2011). Celiac disease is diagnosed using clinical, biochemical, and intestinal biopsy techniques in a GWAS study. We exclusively used SNPs from individuals of European population to limit the bias of population stratification.

### SNP selection

We set a series of quality control criteria to filter and get the eligible SNPs. We selected SNPs that were intensely related to exposure (p < 5E-08) and had independent inheritance without obvious linkage disequilibrium (LD) (r^2^ < 0.001) (kb = 10,000). Moreover, minor allele frequencies (MAF) 0.01 and moderately frequent echo SNPs were eliminated. According to the third central hypothesis, SNPs directly associated with celiac disease and IBD were excluded, which allowing genetic variation to act on outcomes only through exposure. We also used the F-statistic to measure the strength of the instrument; IVs having an F-statistic <10 were deemed “weak instruments” and eliminated. SNPs that passed rigorous selection were used as final instrument SNPs for subsequent MR analysis.

### Effect size estimate

An SNP was selected as an IV based on the above criteria. We assessed the role of exposure in the outcome using the principle of two-sample MR. To explore the causal link between exposure and outcome, we utilized the following four distinct methods: inverse variance weighting (IVW), MR Egger, weighted median (WM), and maximum likelihood ratio. The IVW method obtained the most accurate results by combining the Wald ratios of each SNP. The amount of directional pleiotropy can be estimated through MR-Egger procedure. However, its results may be inaccurate and susceptible to genetic variation (Bowden et al., 2016a). Although the WM method improves accuracy compared to MR-Egger, its accuracy is still suboptimal at <50% of the percentage change in horizontal pleiotropic variation (Bowden et al., 2016b). Probability of distribution parameters were measured using the maximum likelihood ratio approach (Milligan, 2003). The reliability of the results was verified using the MR-Steiger directionality test (Hemani et al., 2017).

### Sensitivity analyses

Distinct sensitivity analyses were performed to establish reliability of the results. The Cochran Q statistic was used to estimate the heterogeneity of the instrumental variables, which were assessed using IVW and WM techniques. The presence of horizontal pleiotropy was determined using the MR-PRESSO test. The “leave-one-out sensitivity analysis” method was executed, which involves removing SNPs one by one and identifying whether the effect of a single SNP seriously distorts the results.

### Bidirectional Mendelian randomization

We reversed the exposure and outcome inputs, thereby performing a bidirectional MR analysis to determine the impact of higher celiac disease genetic risk on IBD, UC, and CD. The SNPs related to celiac disease were derived from the GWAS study data of celiac disease, which included 12,041 cases and 12,228 controls (Trynka et al., 2011). GWAS data from the IBD Genetics Consortium were then utilized as outcomes (Liu et al., 2015). We used the same SNP selection criteria and MR analysis methods as in the bidirectional study. We performed Bonferroni correction to prevent false positive results in two-way multiple testing. After Bonferroni correction, *p*-values < 0.0167 on both sides were considered statistically significant, while *p*-values > 0.0167 and <0.05 were regarded as suggestively significant without multiple testing corrections.

All statistical data were calculated using the two-sample MR program “TwoSampleMR” for R language version 3.6.1.

## Results

Based on the above selection criteria, we included 53, 42, and 43 IV SNPs for IBD, UC, and CD, respectively. The F-values of these instrumental variables were all >10 (ranging from 30.3412 to 274.9761 for IBD, 29.7327 to 191.4428 for UC, and 30.9949 to 349.9935 for CD), with mean F-values of 85.3643, 56.4157, and 80.4624, respectively (Supplementary Tables S1–S3). The results revealed that instrumental bias cannot directly affect the assessment of causal effects.

**FIGURE 1:**
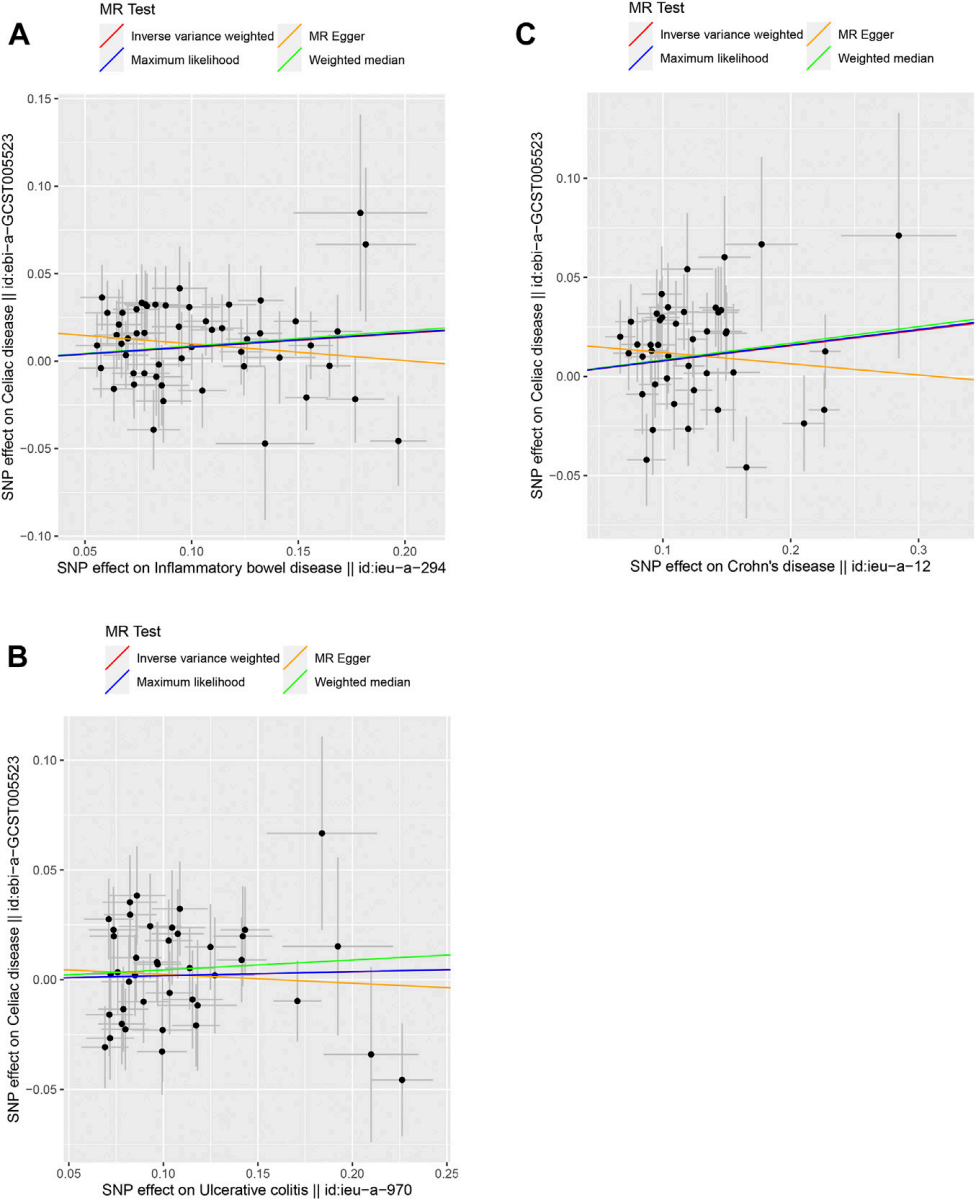
Scatter plots of the genetic causal associations with IBD, UC, and CD against celiac disease using different MR methods. **(A)** IBD against celiac disease; **(B)** UC against celiac disease; and **(C)** CD against celiac disease. The slopes of the line represent the causal association for different methods. The red line represents the Inverse variance weighted (IVW), the yellow line represents the MR-Egger, the blue line represents the Maximum likelihood estimate, and the green line represents the Weighted median.

The causal link between IBD and celiac disease was inconsistent across the four MR methods. The MR results for IVW and WM showed a significant association between IBD and celiac disease (IVW odds ratio (OR) = 1.0828, 95% CI = 1.0258–1.1428, *p* = 0.0039) and WM showed a significant suggestive association (WM OR = 1.0902, 95% CI = 1.0066–1.1809, *p* = 0.0381); however, the MR-Egger method did not show a significant link between IBD and celiac disease (OR = 0.9089, 95% CI = 0.7744–1.0667) (Table 1 and Figure 1). Because the IVW method has higher accuracy than the MR-Egger method and is consistent with WM estimates, we conclude that IBD has a positive causal effect on celiac disease. For UC, none of the methods indicated a significant link with celiac disease (IVW OR = 1.0184, 95% CI = 0.9587–1.0818, *p* = 0.5539; WM OR = 1.0460, 95% CI = 0.9589–1.1411, *p* = 0.3103; MR-Egger OR = 0.9599, 95% CI = 0.7838–1.1756, *p* = 0.6947) (Table 1 and Figure 1). The causal effect of CD on celiac disease was the same as that of IBD, (IVW OR = 1.0807, 95% CI = 1.0227–1.1420, *p* = 0.0058; WM OR = 1.0871, 95% CI = 1.0011–1.1806, *p* = 0.0470; MR-Egger OR = 0.9452, 95% CI = 0.7970–1.1208, *p* = 0.5203) (Table 1 and Figure 1). In addition, the MR-Steiger test supported the results of a causal link between IBD, UC, CD, and celiac disease (Table 1).

**TABLE 1:** MR estimates from each method of assessing the causal effects of inflammatory bowel disease, ulcerative colitis, Crohn’s disease on celiac disease risk.

Exposure traits	MR methods	Celiac disease				
		Number of SNPs	Or (95% CI)	SE	MR *p*-value	MR-steiger test
IBD	MR-Egger	53	0.9089 (0.7744–1.0667)	0.0817	0.2476	Direction: TRUE *p*-value <0.0001
	Inverse variance weighted	53	1.0828 (1.0258–1.1428)	0.0275	0.0039	
	Weighted median	53	1.0902 (1.0066–1.1809)	0.0417	0.0381	
	Maximum likelihood	53	1.0843	0.0276	0.0033	
			(1.0272–1.1445)			
UC	MR-Egger	42	0.9599 (0.7838–1.1756)	0.1034	0.6947	Direction: TRUE *p*-value <0.0001
	Inverse variance weighted	42	1.0184 (0.9587–1.0818)	0.0308	0.5539	
	Weighted median	42	1.0460 (0.9589–1.1411)	0.0444	0.3103	
	Maximum likelihood	42	1.0187 (0.9596 1.0814)	0.0305	0.5441	
CD	MR-Egger	43	0.9452 (0.7970–1.1208 (	0.0870	0.5203	Direction: TRUE *p*-value <0.0001
	Inverse variance weighted	43	1.0807 (1.0227–1.1420)	0.0282	0.0058	
	Weighted median	43	1.0871 (1.0011–1.1806)	0.0421	0.0470	
	Maximum likelihood	43	1.0822 (1.0284–1.1390)	0.0261	0.0024	

MR, mendelian randomization; SNP, single nucleotide polymorphism; IBD, inflammatory bowel disease; UC, ulcerative colitis; CD, Crohn’s disease; OR, odds ratio; CI, confidence interval; SE, standard error.

Heterogeneity, multiplicity, and sensitivity analyses ensured that the final results were of high quality. Cochran Q values indicated no heterogeneity between the IV estimates determined by the MR-Egger and IVW methods (for IBD, MR-Egger Q = 47.4391, *p* = 0.6159; IVW Q = 52.6127, *p* = 0.4502. For UC, MR-Egger Q = 42.2388, *p* = 0.3745; IVW Q = 42.6183, *p* = 0.4013. Egger Q = 42.2388, *p* = 0.3745; IVW Q = 42.6183, *p* = 0.4013. For CD, MR-Egger Q = 46.8851, *p* = 0.2438; IVW Q = 49.9047, *p* = 0.1880.) (Table 2). We used the MR-PRESSO method to test for horizontal pleiotropy and did not discover any bias caused by horizontal pleiotropy (*p*-values for IBD, UC, and CD were 0.4340, 0.3980, and 0.1700, respectively) (Table 2). We performed a “leave-one-out sensitivity analysis” and found that no single SNP strongly influenced the overall effect of IBD, UC, and CD on celiac disease (Supplementary Figures 1–3).

**TABLE 2:** Heterogeneity and pleiotropy analysis of inflammatory bowel disease, ulcerative colitis, Crohn’s disease with celiac disease risk using different analytic methods.

Exposure traits	MR methods	Celiac disease		
		Cochran Q statistic	Heterogeneity p-value	Pleiotropy p-value
IBD	MR-Egger	47.4391	0.6159	0.4340
	Inverse variance weighted	52.6127	0.4502	
UC	MR-Egger	42.2388	0.3745	0.3980
	Inverse variance weighted	42.6183	0.4013	
CD	MR-Egger	46.8851	0.2438	0.1700
	Inverse variance weighted	49.9047	0.1880	

IBD, inflammatory bowel disease; UC, ulcerative colitis; CD, Crohn’s disease.

**FIGURE 2:**
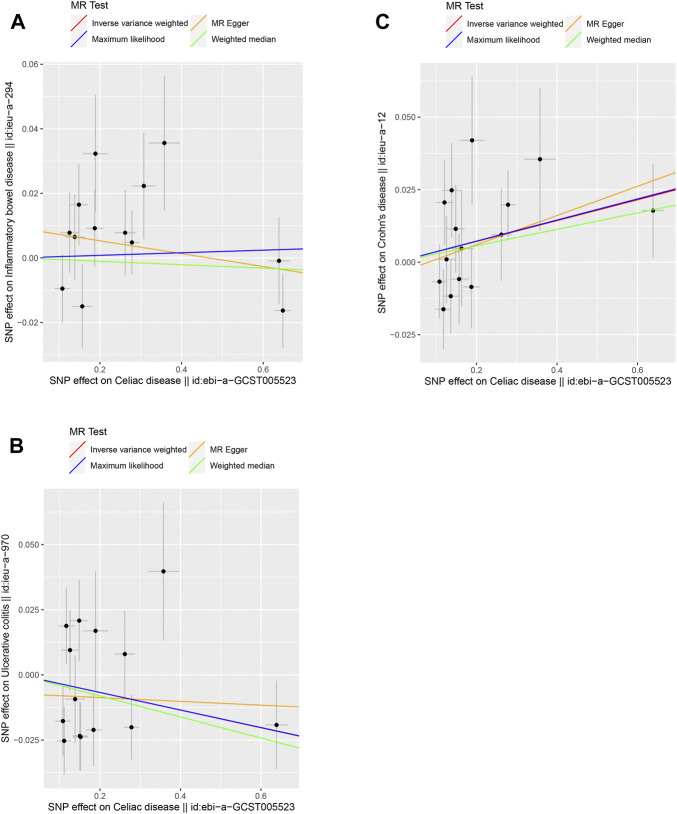
Scatter plots of the genetic causal associations with celiac disease against IBD, UC, and CD using different MR methods. **(A)** Celiac disease against IBD; **(B)** celiac disease against UC; and **(C)** celiac disease against CD. The slopes of the line represent the causal association for different methods. The red line represents the Inverse variance weighted (IVW), the yellow line represents the MR-Egger, the blue line represents the Maximum likelihood estimate, and the green line represents the Weighted median.

We selected 13, 14, and 15 SNPs from the GWAS using the same criteria to explore the causality of celiac disease on IBD, UC, and CD, respectively (Supplementary Tables S4-S6). The F-values of these instrumental variables were all greater than 10 (ranging from 59.6145 to 2072.651 for IBD, 59.6121 to 1,079.617 for UC, and 58.6034 to 1,079.568 for CD), with mean F-values of 367.0868, 199.6983, and 183.2043, respectively (Supplementary Tables S4–S6). There was no strong evidence for a causal link between celiac disease and IBD or UC according to the selected MR method, and Bonferroni corrected significance threshold *p* < 0.0167 (Supplementary Table S7 and Figure 2). For celiac disease and CD, the IVW method showed a possible positive causal relationship between celiac disease and CD (OR = 1.0366, 95% CI = 1.0031–1.0711, *p* = 0.0319) (Supplementary Table S7; Figure 2). Cochran’s Q values indicated no heterogeneity between the IV estimates determined by the MR-Egger and IVW methods (Supplementary Table S8). We did not observe any bias caused by horizontal pleiotropy (Supplementary Table S8). No single SNP strongly influenced the overall outcome of celiac disease on IBD, UC, and CD by using a “leave-one-out” sensitivity analysis.

Overall, our study found that IBD was positively linked with an increased risk of celiac disease. As two subunits of IBD, UC showed no causal relationship with celiac disease in the subunits compared to CD. In the reverse MR analysis, celiac disease did not show an association with IBD and UC, whereas there was a positive link between celiac disease and CD.

## Discussion

MR analysis and a large sample of GWAS data were used to explain the bidirectional causal link between IBD (including UC and CD) and celiac disease. Our data showed a causal link between IBD (UC and CD) and celiac disease, indicating that their etiologies might be similar.

Researchers have been studied that IBD and celiac disease have the similar genetic background; IL18RAP, PTPN2, TAGAP, and PUS10 have been identified as shared risk loci for both CD and celiac disease (Glas et al., 2009; Festen et al., 2011). In IBD and celiac disease, both of which are related to increased intestinal penetration, gut wall function is crucial (Camilleri et al., 2012; Cardoso-Silva et al., 2019). T regulatory cells (Hmida et al., 2012; Hisamatsu et al., 2016) and cytokines (IL-15, IL-17, and IL-21) (Jabri and Abadie, 2015; Meisel et al., 2017) are implicated in immunological responses in both illnesses. Related reviews have also reported a mechanistic link between the gut microbiota and these two diseases (Harris and Chang, 2018; Caminero et al., 2019). The genetic, immunological, and intestinal environmental variables indicate that IBD and celiac disease have the similar pathophysiology. These findings also suggest a possible link between these two disorders.

There is growing evidence that the gut microbiota is strongly associated with autoimmune diseases including IBD and celiac disease (Chen et al., 2017; De Luca and Shoenfeld, 2019). Bifidobacterium suppresses IBD by modulating cup cell stress (Engevik et al., 2021). In addition, the reduction in bifidobacteria activates the inflammatory response of gluten in celiac disease patients (Medina et al., 2008). The causal relationship between the gut microbiota and IBD and celiac disease has been reported in the literature (Garcia-Santisteban et al., 2020; Zhang et al., 2021). In the future, the use of two-step MR and multivariable MR to further explore the role of intestinal flora and its metabolites in the pathogenesis of these two diseases is a novel research direction.

For valid causal deduction, it is essential to ensure that the intermediate “exposure” or intermediate phenotypes appear before the succeeding “disease outcome.” Some previous observational studies have demonstrated the tempo order between IBD and celiac disease (Tursi et al., 2005; Yang et al., 2005; Casella et al., 2010). Future studies should further investigate the timing of disease events to determine the temporal sequence of exposure versus disease outcomes. Several previous meta-analyses have observed a positive causal relationship between IBD and celiac disease, with a higher risk of IBD in patients with CD than in patients with IBD, with a higher risk of CD than UC(5, 33). This is not completely consistent with our results because of the confounding issues in observational research. Our data showed that IBD is associated with a higher risk of celiac disease. Regarding UC and CD, the risk of celiac disease in CD patients has statistical significance. Conversely, we only found a suggestive positive correlation between celiac disease and CD.

Evidence of a causal link between IBD and celiac disease may aid in the development of better screening tools and therapeutic management. Different clinical management strategies may be undertaken for IBD patients with or without celiac disease. IBD patients who also have celiac disease are more likely to develop unfavorable conditions (extensive colitis and primary sclerosing cholangitis); hence, screening for celiac disease in IBD patients may be beneficial. Celiac disease depends on serological testing and duodenal biopsies for an accurate diagnosis according to US and European criteria (Rubio-Tapia et al., 2013; Ludvigsson et al., 2014). Due to the invasive and expensive nature of duodenal biopsy collection, we propose that individuals diagnosed with IBD undergo regular serological testing to monitor the possibility of celiac disease. Further research is required to determine whether screening for IBD in celiac disease is beneficial. As a stimulus for celiac disease, gluten is a hot topic in the dietary management of IBD patients (Weaver and Herfarth, 2021). Based on our study, a low-gluten diet in the dietary management of patients with IBD is of recommended value. Studies have revealed that the clinical expressions of celiac disease and IBD are potentially similar (Green, 2005; Rampertab et al., 2006). These findings provide doctors additional diagnostic and treatment options. Clinicians should reconsider IBD or celiac disease as a secondary diagnosis when patients do not react well to medication (Pinto-Sanchez et al., 2020). Specifying the causal link between IBD and celiac disease will also shed fresh light on research investigating the pathogenesis of the illness.

Our study has certain limitations. First, the research population for exposure and results had European ancestry, which declined population stratification but produced no typical findings of people of different ancestries. Second, we did not stratify the causal relationship between IBD and celiac disease by sex or age, which could potentially affect the study results. Third, although we performed many sensitivity analyses, we could not guarantee that every SNP met the three IV conditions. We examined known confounders such as smoking and obesity and eliminated potentially relevant IVs. However, unknown confounding factors might have unavoidably affected causal inference. In addition, the MR analysis reflected lifetime exposure; however, it was not possible to determine how specific exposure times affected the results. Finally, we do not propose a mechanism to explain the causal link between IBD and celiac disease. Further studies are required to investigate the underlying mechanism that may improve disease prevention.

In conclusion, we report that IBD was positively linked with an increased risk of celiac disease, while UC did not show a causal association with celiac disease compared to CD. Furthermore, in the reverse MR analysis, celiac disease did not show an association with IBD and UC; however, there was a positive link between celiac disease and CD.

## Data Availability

The datasets presented in this study can be found in online repositories. The names of the repository/repositories and accession number(s) can be found in the article/Supplementary Material.
