# Supplementary material for: Bidirectional causal link between inflammatory bowel disease and celiac disease: A two-sample mendelian randomization analysis
Source: Front Genet. 2022 Sep 20;13:993492. doi: 10.3389/fgene.2022.993492 (PMC9530974; doi:10.3389/fgene.2022.993492)
Supplement: Supplementary file 1 [file DataSheet1.PDF]

Table S1. Characteristics of the SNPs used in the Mendelian randomization analysis of the effects of inflammatory bowel disease on celiac disease risk.
Table S2. Characteristics of the SNPs used in the Mendelian randomization analysis of the effects of ulcerative colitis on celiac disease risk.
Table S3. Characteristics of the SNPs used in the Mendelian randomization analysis of the effects of Crohn’s disease on celiac disease risk.
Table S4. Characteristics of the SNPs used in the Mendelian randomization analysis of the effects of celiac disease on inflammatory bowel disease risk.
Table S5. Characteristics of the SNPs used in the Mendelian randomization analysis of the effects of celiac disease on ulcerative colitis risk.
Table S6. Characteristics of the SNPs used in the Mendelian randomization analysis of the effects of celiac disease on Crohn’s disease risk.
Table S7. MR estimates from each method of assessing the causal effects of celiac disease on IBD, ulcerative colitis and Crohn’s disease risk.
Table S8. Heterogeneity and pleiotropy analysis of celiac disease with IBD, ulcerative colitis and Crohn’s disease risk using different analytic methods.
Figure S1. “Leave-one-out” sensitivity analysis of causal effects of inflammatory bowel disease on celiac disease.
Figure S2. “Leave-one-out” sensitivity analysis of causal effects of ulcerative colitis on celiac disease.
Figure S3. “Leave-one-out” sensitivity analysis of causal effects of Crohn’s disease on celiac disease.
Figure S4. “Leave-one-out” sensitivity analysis of causal effects of celiac disease on inflammatory bowel disease.
Figure S5. “Leave-one-out” sensitivity analysis of causal effects of celiac disease on ulcerative colitis.
Figure S6. “Leave-one-out” sensitivity analysis of causal effects of celiac disease on Crohn’s disease.

Targe SNPs	Chr	Effect allele*	Other allele*	Association with IBD				Association with celiac disease		
				β	SE	p-Value	F-Statistic	β	SE	p-Value
rs4692386	4	C	T	0.0580	0.0102	1.21E-08	32.4423	0.0364	0.0188	0.0533
rs3024493	1	A	C	0.1969	0.0132	1.65E-50	223.2183	-0.0456	0.0257	0.0755
rs7848647	9	C	T	0.1324	0.0107	3.16E-35	153.2645	0.0347	0.0198	0.0796
rs559928	11	C	T	0.0944	0.0130	3.33E-13	52.9645	0.0416	0.0240	0.0834
rs1267499	6	C	T	0.0821	0.0125	5.22E-11	43.0613	-0.0392	0.0228	0.0857
rs780094	2	C	T	-0.0783	0.0100	3.88E-15	61.7101	-0.0325	0.0192	0.0913
rs11677953	2	A	G	0.0791	0.0100	2.92E-15	62.2699	0.0315	0.0187	0.0917
rs6500315	16	G	A	0.0766	0.0119	1.12E-10	41.5663	0.0334	0.0219	0.1286
rs2395022	7	C	A	-0.1816	0.0234	8.27E-15	60.2236	-0.0667	0.0440	0.1293
rs941823	13	C	T	0.0830	0.0115	6.19E-13	51.7464	0.0323	0.0215	0.1320
rs62434177	6	A	G	-0.1791	0.0314	1.14E-08	32.5619	-0.0847	0.0562	0.1321
rs2274351	10	T	C	0.0605	0.0104	6.93E-09	33.5284	0.0276	0.0184	0.1337
rs2297559	1	A	G	0.0742	0.0110	1.88E-11	45.0514	0.0296	0.0201	0.1394
rs2538470	7	G	A	-0.0676	0.0102	3.00E-11	44.1428	-0.0276	0.0190	0.1459
rs9557207	13	G	A	-0.0878	0.0121	3.52E-13	52.8555	-0.0318	0.0224	0.1560
rs35164067	19	A	G	-0.1175	0.0127	2.66E-20	85.1636	-0.0323	0.0231	0.1622
rs12722515	10	A	C	-0.0989	0.0143	4.57E-12	47.8272	-0.0309	0.0259	0.2326
rs34779708	10	G	T	0.1067	0.0102	2.07E-25	108.4308	0.0227	0.0196	0.2450
rs10758669	9	A	C	-0.1488	0.0102	4.70E-48	211.9687	-0.0227	0.0196	0.2466
rs10761659	10	G	A	0.1538	0.0100	4.97E-53	234.7623	-0.0208	0.0188	0.2681
rs7253253	19	T	G	-0.1344	0.0231	6.19E-09	33.7493	0.0471	0.0437	0.2811
rs1182188	7	C	T	-0.0659	0.0108	1.08E-09	37.1476	-0.0209	0.0203	0.3019
rs8127691	21	C	T	-0.1143	0.0101	8.98E-30	128.3415	-0.0188	0.0192	0.3258

rs12103	1	C	T	-0.0867	0.0131	3.28E-11	43.9682	0.0229	0.0237	0.3342
rs12318183	12	A	C	0.1095	0.0101	1.67E-27	117.9819	0.0178	0.0188	0.3416
rs2143178	22	C	T	-0.1767	0.0137	4.80E-38	166.1529	0.0218	0.0248	0.3802
rs2328546	6	C	T	0.0940	0.0127	1.30E-13	54.8131	0.0197	0.0231	0.3941
rs1517352	2	C	A	0.0779	0.0103	3.87E-14	57.1913	0.0161	0.0189	0.3943
rs2497318	10	T	C	-0.0635	0.0099	1.36E-10	41.1914	0.0159	0.0188	0.3975
rs10800309	1	G	A	-0.1321	0.0104	6.15E-37	161.0854	-0.0159	0.0192	0.4093
rs2836883	21	A	G	-0.1684	0.0115	3.38E-48	212.6268	-0.0169	0.0210	0.4192
rs9889296	17	A	G	-0.1050	0.0113	1.35E-20	86.4965	0.0169	0.0212	0.4265
rs648541	11	G	A	-0.0649	0.0107	1.22E-09	36.9129	-0.0149	0.0195	0.4449
rs6074022	20	T	C	-0.0743	0.0114	8.32E-11	42.1479	-0.0159	0.0209	0.4475
rs1292053	17	G	A	0.0701	0.0098	9.89E-13	50.8266	0.0129	0.0182	0.4776
rs4976646	5	C	T	0.0730	0.0105	3.23E-12	48.5031	-0.0134	0.0194	0.4904
rs7194886	16	T	C	-0.1260	0.0100	2.53E-36	158.2744	-0.0126	0.0188	0.5041
rs2153283	10	A	C	-0.0860	0.0127	1.54E-11	45.4526	0.0139	0.0231	0.5467
rs1990760	2	T	C	-0.0671	0.0107	3.56E-10	39.3085	-0.0100	0.0197	0.6136
rs6058869	20	T	C	0.0557	0.0100	2.63E-08	30.9351	0.0090	0.0180	0.6196
rs56167332	5	A	C	0.1559	0.0105	7.17E-50	220.2917	0.0090	0.0193	0.6424
rs744166	17	G	A	-0.1000	0.0102	1.14E-22	95.9318	-0.0080	0.0187	0.6673
rs17780256	17	C	A	-0.0834	0.0126	3.19E-11	44.0203	0.0090	0.0223	0.6877
rs974801	4	G	A	-0.0728	0.0101	7.07E-13	51.4833	0.0070	0.0187	0.7096
rs2270395	16	T	C	0.0778	0.0119	5.17E-11	43.0772	-0.0070	0.0217	0.7479
rs6062496	20	A	G	0.1232	0.0102	2.11E-33	144.9251	0.0053	0.0188	0.7771
rs34804116	5	A	C	-0.0575	0.0104	3.62E-08	30.3172	0.0040	0.0169	0.8137
rs7523442	1	T	C	0.1245	0.0099	2.76E-36	158.1012	-0.0030	0.0167	0.8578
rs913678	20	C	T	-0.0692	0.0105	5.35E-11	43.0127	-0.0034	0.0195	0.8614

rs6584281	10	G	A	-0.1646	0.0099	9.36E-62	274.7583	0.0027	0.0181	0.8814
rs55808324	14	A	G	0.1412	0.0168	5.13E-17	70.2297	0.0020	0.0264	0.9397
rs79980175	5	C	A	-0.0953	0.0148	1.30E-10	41.2703	-0.0016	0.0264	0.9517
rs7011507	8	A	G	-0.0846	0.0151	2.03E-08	31.4389	0.0020	0.0364	0.9562

SNP=single nucleotide polymorphism; Chr = chromosome; IBD = inflammatory bowel disease; β = regression effect size; SE= standard error (the standard error is an estimate of the standard deviation (SD) of the coefficient)

Table S1. Characteristics of the SNPs used in the Mendelian randomization analysis of the effects of inflammatory bowel disease on celiac disease risk.

Targe SNPs	Chr	Effect allele	Other allele	Association with ulcerative colitis				Association with celiac disease		
				β	SE	p-Value	F-Statistic	β	SE	p-Value
rs3024493	1	A	C	0.2263	0.0163	1.42E-43	191.4428	-0.0456	0.0257	0.0755
rs16841904	1	T	C	0.0860	0.0153	1.90E-08	31.5641	0.0383	0.0225	0.0886
rs3774937	4	C	T	0.0993	0.0132	4.61E-14	56.8426	-0.0327	0.0197	0.0969
rs11229555	11	T	G	-0.0823	0.0145	1.21E-08	32.4361	-0.0353	0.0214	0.0983
rs11083840	19	G	T	0.0691	0.0125	3.41E-08	30.4334	-0.0307	0.0187	0.1009
rs2395022	7	C	A	-0.1839	0.0292	2.88E-10	39.7216	-0.0667	0.0440	0.1293
rs941823	13	C	T	0.1087	0.0147	1.39E-13	54.6724	0.0323	0.0215	0.1320
rs2274351	10	T	C	0.0711	0.0130	4.90E-08	29.7327	0.0276	0.0184	0.1337
rs4656958	1	G	A	0.0824	0.0139	2.82E-09	35.2770	0.0296	0.0200	0.1387
rs7404095	16	C	T	0.0718	0.0127	1.52E-08	31.9955	-0.0266	0.0189	0.1593
rs11150589	16	C	T	-0.0799	0.0127	3.28E-10	39.4649	0.0226	0.0185	0.2236
rs10758669	9	A	C	-0.1432	0.0129	1.04E-28	123.4840	-0.0227	0.0196	0.2466
rs59418206	10	A	G	0.0736	0.0130	1.45E-08	32.0923	0.0227	0.0197	0.2486
rs10761659	10	G	A	0.1173	0.0126	1.50E-20	86.2905	-0.0208	0.0188	0.2681
rs11641184	16	A	C	0.0780	0.0125	4.24E-10	38.9661	-0.0201	0.0185	0.2781
rs4747886	10	T	C	0.0738	0.0129	9.58E-09	32.8964	0.0198	0.0187	0.2893
rs1182188	7	C	T	-0.1076	0.0138	5.03E-15	61.1972	-0.0209	0.0203	0.3019
rs4712520	6	C	T	0.0930	0.0166	2.21E-08	31.2757	0.0244	0.0240	0.3101
rs12103	1	C	T	-0.0996	0.0163	9.96E-10	37.3008	0.0229	0.0237	0.3342
rs4456788	21	A	G	-0.1028	0.0127	7.07E-16	65.0594	-0.0178	0.0187	0.3397
rs76904798	12	T	C	0.1046	0.0176	2.78E-09	35.3028	0.0237	0.0261	0.3632
rs4676410	2	A	G	0.1420	0.0157	1.85E-19	81.3194	0.0198	0.0228	0.3859
rs34659678	6	T	C	0.2099	0.0251	5.95E-17	69.9339	-0.0340	0.0399	0.3941
rs2497318	10	T	C	-0.0714	0.0125	1.15E-08	32.5484	0.0159	0.0188	0.3975

rs661054	11	G	A	-0.1249	0.0136	3.18E-20	84.8016	-0.0149	0.0195	0.4448
rs4976646	5	C	T	0.0788	0.0132	2.52E-09	35.4958	-0.0134	0.0194	0.4904
rs1801274	1	G	A	-0.1709	0.0127	1.43E-41	182.2689	0.0097	0.0184	0.5968
rs9891119	17	C	A	-0.0895	0.0133	1.72E-11	45.2243	0.0100	0.0189	0.5993
rs1990760	2	T	C	-0.0856	0.0134	1.78E-10	40.6595	-0.0100	0.0197	0.6136
rs56167332	5	A	C	0.1414	0.0132	7.27E-27	115.0595	0.0090	0.0193	0.6424
rs10185424	2	G	T	-0.0966	0.0126	1.47E-14	59.0828	-0.0080	0.0183	0.6630
rs17780256	17	C	A	-0.1154	0.0160	6.13E-13	51.7620	0.0090	0.0223	0.6877
rs79045992	16	A	G	0.1181	0.0208	1.43E-08	32.1148	-0.0117	0.0298	0.6957
rs111830527	1	A	G	-0.1923	0.0293	5.09E-11	43.1071	-0.0152	0.0405	0.7074
rs4728142	7	A	G	0.0970	0.0127	1.92E-14	58.5652	0.0070	0.0192	0.7157
rs4812833	20	A	G	0.1033	0.0126	1.87E-16	67.6775	-0.0060	0.0190	0.7526
rs6062496	20	A	G	0.1139	0.0129	9.14E-19	78.1710	0.0053	0.0188	0.7771
rs913678	20	C	T	-0.0758	0.0133	1.23E-08	32.4133	-0.0034	0.0195	0.8614
rs1077773	7	A	G	0.0721	0.0124	5.96E-09	33.8176	0.0025	0.0187	0.8935
rs483905	11	A	G	0.0850	0.0135	3.16E-10	39.5406	0.0020	0.0185	0.9139
rs55808324	14	A	G	0.1272	0.0210	1.47E-09	36.5415	0.0020	0.0264	0.9397
rs10460566	2	A	G	-0.0818	0.0145	1.60E-08	31.9054	0.0009	0.0214	0.9664

SNP=single nucleotide polymorphism; Chr = chromosome; β = regression effect size; SE= standard error (the standard error is an estimate of the standard deviation (SD) of the coefficient)

Table S2. Characteristics of the SNPs used in the Mendelian randomization analysis of the effects of ulcerative colitis on celiac disease risk.

Targe SNPs	Chr	Effect allele	Other allele	Association with Crohn's disease				Association with celiac disease		
				β	SE	p-Value	F-Statistic	β	SE	p-Value
rs61839660	10	T	C	0.1482	0.0203	3.19E-13	53.0876	0.0602	0.0310	0.0522
rs17391694	1	T	C	-0.1191	0.0200	2.62E-09	35.4498	-0.0541	0.0283	0.0556
rs1267501	6	C	T	0.0872	0.0152	9.69E-09	32.9028	-0.0421	0.0233	0.0704
rs3024505	1	A	G	0.1653	0.0160	3.95E-25	107.2410	-0.0459	0.0257	0.0735
rs7848647	9	C	T	0.1413	0.0130	1.55E-27	118.2267	0.0347	0.0198	0.0796
rs559928	11	C	T	0.0991	0.0158	3.75E-10	39.2418	0.0416	0.0240	0.0834
rs780094	2	C	T	-0.1165	0.0121	4.56E-22	93.2738	-0.0325	0.0192	0.0913
rs6908425	6	C	T	0.1040	0.0150	4.81E-12	47.7650	0.0349	0.0224	0.1198
rs6500315	16	G	A	0.1455	0.0146	2.18E-23	99.2952	0.0334	0.0219	0.1286
rs2395022	7	C	A	-0.1772	0.0282	3.13E-10	39.5895	-0.0667	0.0440	0.1293
rs2538470	7	G	A	-0.0750	0.0123	1.05E-09	37.2382	-0.0276	0.0190	0.1459
rs1456896	7	T	C	0.0977	0.0131	1.03E-13	55.3008	0.0284	0.0200	0.1551
rs26528	16	C	T	0.1198	0.0122	1.29E-22	95.7672	-0.0265	0.0187	0.1559
rs9554587	13	G	A	-0.0952	0.0147	8.29E-11	42.1891	-0.0317	0.0224	0.1567
rs2227551	10	T	G	0.0994	0.0137	4.72E-13	52.3171	0.0298	0.0211	0.1570
rs35164067	19	A	G	-0.1434	0.0156	3.19E-20	84.8704	-0.0323	0.0231	0.1622
rs6702421	1	T	C	0.1102	0.0141	6.53E-15	60.7374	0.0266	0.0216	0.2182
rs727563	22	T	C	-0.0921	0.0145	1.88E-10	40.5852	0.0270	0.0228	0.2363
rs34779708	10	G	T	0.1345	0.0124	1.90E-27	117.8226	0.0227	0.0196	0.2450
rs10758669	9	A	C	-0.1498	0.0123	4.19E-34	148.2526	-0.0227	0.0196	0.2466
rs17129991	1	T	C	-0.2845	0.0451	2.81E-10	39.8007	-0.0711	0.0619	0.2511
rs915286	13	A	G	0.0666	0.0120	2.59E-08	30.9949	0.0200	0.0186	0.2826
rs8127691	21	C	T	-0.1234	0.0122	4.48E-24	102.4286	-0.0188	0.0192	0.3258

rs2413583	22	T	C	-0.2104	0.0168	7.72E-36	156.1872	0.0237	0.0242	0.3270
rs11117431	16	G	A	-0.1493	0.0164	1.09E-19	82.4382	-0.0218	0.0241	0.3641
rs6738490	2	C	T	0.2262	0.0121	4.26E-78	349.9935	-0.0169	0.0188	0.3703
rs1517352	2	C	A	0.0800	0.0125	1.31E-10	41.2892	0.0161	0.0189	0.3943
rs10800309	1	G	A	-0.0904	0.0126	8.48E-13	51.1693	-0.0159	0.0192	0.4093
rs9889296	17	A	G	-0.1431	0.0138	2.96E-25	107.8143	0.0169	0.0212	0.4265
rs6074022	20	T	C	-0.0963	0.0138	2.70E-12	48.8989	-0.0159	0.0209	0.4475
rs1292053	17	G	A	0.0912	0.0119	1.75E-14	58.7991	0.0129	0.0182	0.4776
rs7194886	16	T	C	-0.2270	0.0122	1.42E-77	347.5966	-0.0126	0.0188	0.5041
rs7969592	12	G	A	-0.0732	0.0120	1.04E-09	37.2562	-0.0117	0.0186	0.5294
rs2153283	10	A	C	-0.1088	0.0155	2.39E-12	49.1319	0.0139	0.0231	0.5467
rs12949918	17	C	T	-0.1042	0.0124	3.47E-17	71.0569	-0.0103	0.0188	0.5852
rs35320439	2	C	T	0.0841	0.0138	9.89E-10	37.3461	0.0100	0.0199	0.6172
rs7438704	4	G	A	0.0839	0.0127	3.42E-11	43.9215	-0.0090	0.0201	0.6552
rs2270395	16	T	C	0.1243	0.0145	8.93E-18	73.7383	-0.0070	0.0217	0.7479
rs6062496	20	A	G	0.1201	0.0124	3.82E-22	93.6265	0.0053	0.0188	0.7771
rs34804116	5	A	C	-0.0939	0.0127	1.27E-13	54.8964	0.0040	0.0169	0.8137
rs2284553	21	G	A	0.1032	0.0123	5.63E-17	70.1048	-0.0010	0.0156	0.9489
rs79980175	5	C	A	-0.1343	0.0182	1.70E-13	54.3306	-0.0016	0.0264	0.9517
rs11159833	14	T	C	0.1552	0.0208	7.59E-14	55.9093	0.0020	0.0347	0.9541

SNP=single nucleotide polymorphism; Chr = chromosome; β = regression effect size; SE= standard error (the standard error is an estimate of the standard deviation (SD) of the coefficient)

Table S3. Characteristics of the SNPs used in the Mendelian randomization analysis of the effects of Crohn’s disease on celiac disease risk.

Targe SNPs	Chr	Effect allele	Other allele	Association with celiac disease				Association with IBD		
				β	SE	p-Value	F-Statistic	β	SE	p-Value
rs2499714	6	T	C	0.1890	0.0305	6.08E-10	75.2319	0.0323	0.0182	0.0767
rs9258302	6	C	T	-0.3578	0.0388	2.61E-20	167.5013	-0.0356	0.0208	0.0870
rs1431403	6	C	T	0.6482	0.0200	1.00E-200	2072.6505	-0.0163	0.0116	0.1600
rs76830965	3	A	C	0.3075	0.0284	2.57E-27	230.2786	0.0223	0.0165	0.1768
rs7104791	11	C	T	-0.1484	0.0221	1.89E-11	88.5619	-0.0165	0.0126	0.1900
rs12068671	1	C	T	-0.1567	0.0244	1.40E-10	80.8720	0.0150	0.0130	0.2471
rs61579022	3	A	G	0.1080	0.0188	9.92E-09	64.5454	-0.0095	0.0105	0.3675
rs55743914	6	T	C	0.1873	0.0212	1.14E-18	152.8165	0.0092	0.0119	0.4354
rs10947460	6	A	G	-0.1255	0.0228	3.61E-08	59.6145	-0.0078	0.0124	0.5325
rs1323292	1	A	G	0.2618	0.0253	4.23E-25	210.3901	0.0078	0.0132	0.5560
rs2030519	3	A	G	0.2783	0.0189	3.00E-49	427.4880	0.0048	0.0099	0.6295
rs11801183	1	T	C	-0.1383	0.0245	1.69E-08	62.5187	-0.0065	0.0134	0.6297
rs9268303	6	A	G	-0.6387	0.0272	1.54E-121	1079.6591	0.0009	0.0135	0.9483

SNP=single nucleotide polymorphism; Chr = chromosome; IBD = inflammatory bowel disease; β = regression effect size; SE= standard error (the standard error is an estimate of the standard deviation (SD) of the coefficient)

Table S4. Characteristics of the SNPs used in the Mendelian randomization analysis of the effects of celiac disease on inflammatory bowel disease risk.

Targe SNPs	Chr	Effect allele	Other allele	Association with celiac disease				Association with ulcerative colitis		
				β	SE	p-Value	F-Statistic	β	SE	p-Value
rs1050976	6	T	C	-0.1110	0.0185	1.84E-09	70.9882	0.0253	0.0130	0.0512
rs1018326	2	C	T	0.1519	0.0186	3.06E-16	131.1455	-0.0238	0.0130	0.0676
rs182429	6	G	A	-0.1501	0.0187	8.49E-16	127.2004	0.0234	0.0131	0.0741
rs2030519	3	A	G	0.2783	0.0189	3.00E-49	427.4713	-0.0201	0.0125	0.1079
rs2097282	3	T	C	-0.1840	0.0197	1.13E-20	170.7436	0.0211	0.0139	0.1279
rs9258302	6	C	T	-0.3578	0.0388	2.61E-20	167.4947	-0.0397	0.0265	0.1338
rs61579022	3	A	G	0.1080	0.0188	9.92E-09	64.5428	-0.0177	0.0132	0.1795
rs7104791	11	C	T	-0.1484	0.0221	1.89E-11	88.5585	-0.0208	0.0157	0.1853
rs744254	10	A	G	0.1164	0.0210	3.04E-08	60.2747	0.0188	0.0146	0.1986
rs9268303	6	A	G	-0.6387	0.0272	1.54E-121	1079.6170	0.0192	0.0170	0.2581
rs2499714	6	T	C	0.1890	0.0305	6.08E-10	75.2290	0.0169	0.0228	0.4573
rs10947460	6	A	G	-0.1255	0.0228	3.61E-08	59.6121	-0.0095	0.0155	0.5401
rs11801183	1	T	C	-0.1383	0.0245	1.69E-08	62.5163	0.0093	0.0168	0.5807
rs1323292	1	A	G	0.2618	0.0253	4.23E-25	210.3819	0.0080	0.0166	0.6313

SNP=single nucleotide polymorphism; Chr = chromosome; β = regression effect size; SE= standard error (the standard error is an estimate of the standard deviation (SD) of the coefficient)

Table S5. Characteristics of the SNPs used in the Mendelian randomization analysis of the effects of celiac disease on ulcerative colitis risk.

Targe SNPs	Chr	Effect allele	Other allele	Association with celiac disease				Association with osteoporosis		
				β	SE	p-Value	F-Statistic	β	SE	p-Value
rs2499714	6	T	C	0.1890	0.0305	6.08E-10	75.2256	0.0420	0.0220	0.0558
rs2030519	3	A	G	0.2783	0.0189	3.00E-49	427.4521	0.0198	0.0120	0.0988
rs11801183	1	T	C	-0.1383	0.0245	1.69E-08	62.5134	-0.0248	0.0163	0.1279
rs9258302	6	C	T	-0.3578	0.0388	2.61E-20	167.4872	-0.0355	0.0246	0.1501
rs11851414	14	C	T	0.1204	0.0221	4.71E-08	58.6034	0.0206	0.0146	0.1586
rs1378938	15	C	T	-0.1178	0.0204	7.79E-09	65.4625	0.0162	0.0137	0.2391
rs9268303	6	A	G	-0.6387	0.0272	1.54E-121	1079.5683	-0.0178	0.0161	0.2677
rs4445406	1	C	T	-0.1360	0.0197	5.42E-12	93.3577	0.0117	0.0128	0.3612
rs7104791	11	C	T	-0.1484	0.0221	1.89E-11	88.5545	-0.0115	0.0152	0.4508
rs1323292	1	A	G	0.2618	0.0253	4.23E-25	210.3724	0.0095	0.0159	0.5517
rs55743914	6	T	C	0.1873	0.0212	1.14E-18	152.8037	-0.0085	0.0143	0.5549
rs61579022	3	A	G	0.1080	0.0188	9.92E-09	64.5399	-0.0067	0.0128	0.5989
rs12068671	1	C	T	-0.1567	0.0244	1.40E-10	80.8652	0.0058	0.0157	0.7126
rs79758729	7	G	A	0.1630	0.0291	2.12E-08	61.6493	0.0047	0.0201	0.8163
rs10947460	6	A	G	-0.1255	0.0228	3.61E-08	59.6094	-0.0010	0.0151	0.9444

SNP=single nucleotide polymorphism; Chr = chromosome; β = regression effect size; SE= standard error (the standard error is an estimate of the standard deviation (SD) of the coefficient)

Table S6. Characteristics of the SNPs used in the Mendelian randomization analysis of the effects of celiac disease on Crohn's disease risk.

Exposure traits	MR method	IBD			UC			CD		
		OR (95% CI)	SE	MR p-value	OR (95% CI)	SE	MR p-value	OR (95% CI)	SE	MR p-value
Celiac disease	MR-Egger	0.9803(0.9393~1.0232)	0.0218	0.3824	0.9928(0.9136~1.0789)	0.0424	0.8684	1.0516(0.9897~1.1173)	0.0309	0.1282
	Inverse variance weighted	1.0040(0.9796~1.0290)	0.0125	0.7517	0.9667(0.9250~1.0104)	0.0225	0.1334	1.0366(1.0031~1.0711)	0.0167	0.0319
	Weighted median	0.9948(0.9668~1.0236)	0.0145	0.7201	0.9605(0.9158~1.0073)	0.0243	0.0966	1.0286(0.9842~1.0751)	0.0225	0.2105
	Maximum likelihood	1.0040(0.9824~1.0260)	0.0111	0.7199	0.9669(0.9341~1.0008)	0.0176	0.0554	1.0371(1.0034~1.0719)	0.0168	0.0306

MR = Mendelian randomization; IBD = inflammatory bowel disease; UC = ulcerative colitis; CD = Crohn's disease OR = odds ratio; CI = confidence interval; SE = standard error (the standard error is an estimate of the standard deviation (SD) of the coefficient)

Table S7. MR estimates from each method of assessing the causal effects of celiac disease on IBD, ulcerative colitis and Crohn's disease risk.

Exposure traits	MR methods	inflammatory bowel disease			ulcerative colitis			Crohn's disease		
		Cochran Q statistic	Heterogeneity p-value	Pleiotropy p-value	Cochran Q statistic	Heterogeneity p-value	Pleiotropy p-value	Cochran Q statistic	Heterogeneity p-value	Pleiotropy p-value
Celiac disease	MR-Egger	13.3191	0.2730	0.2280	20.7169	0.0547	0.1230	13.0406	0.4447	0.5950
	Inverse variance weighted	15.4165	0.2194		21.6794	0.0605		13.3468	0.4994	

MR = Mendelian randomization;

Table S8. Heterogeneity and pleiotropy analysis of celiac disease with inflammatory bowel disease, ulcerative colitis and Crohn's disease risk using different analytic methods.

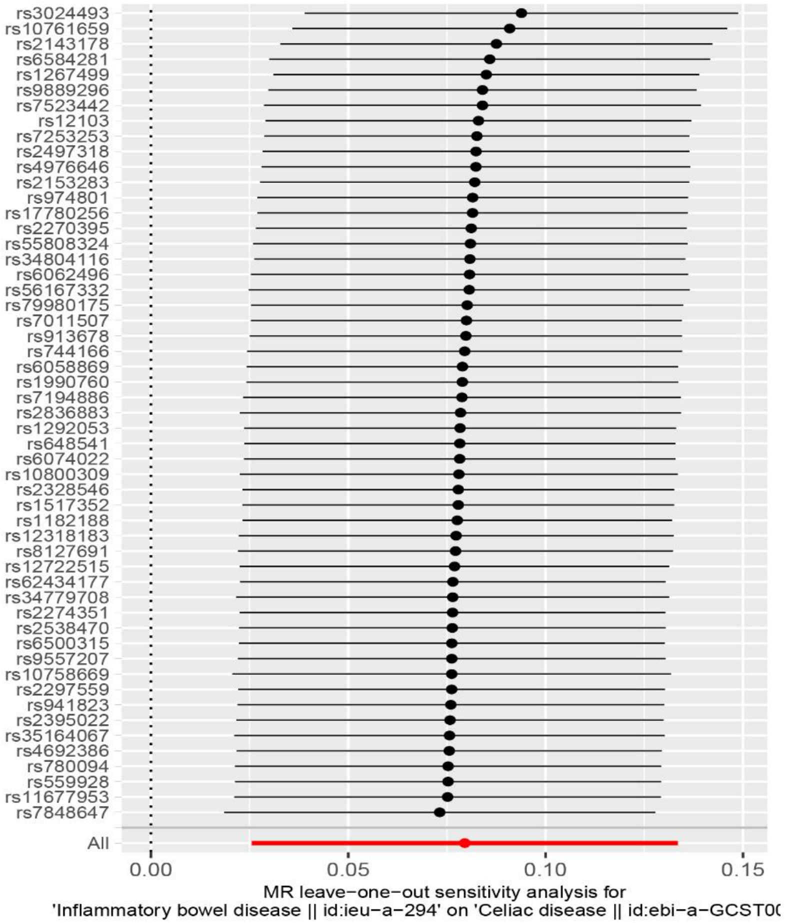

Figure S1. “Leave-one-out” sensitivity analysis of causal effects of inflammatory bowel disease on celiac disease.

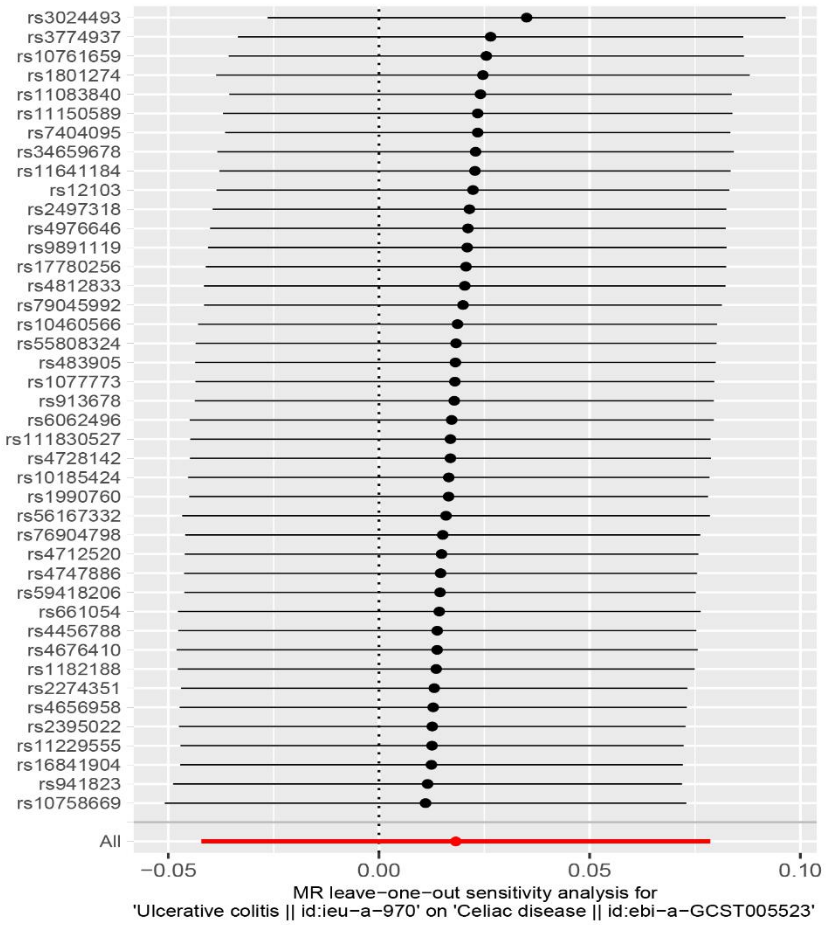

Figure S2. “Leave-one-out” sensitivity analysis of causal effects of ulcerative colitis on celiac disease.

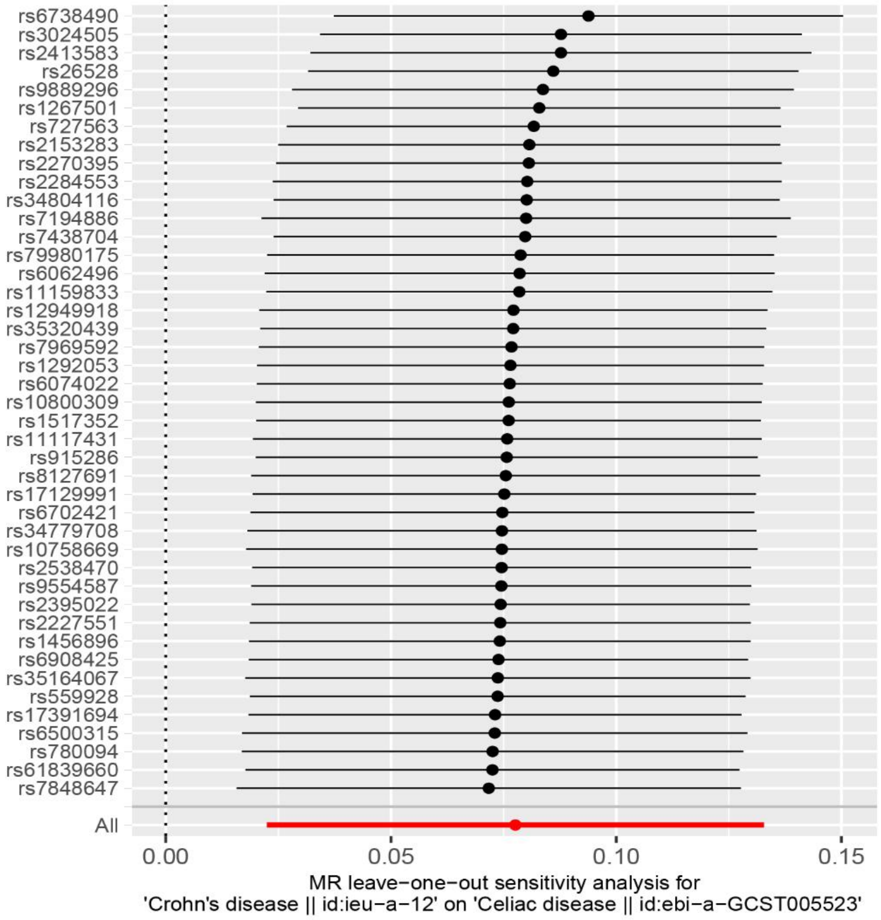

Figure S3. "Leave-one-out" sensitivity analysis of causal effects of Crohn's disease on celiac disease.

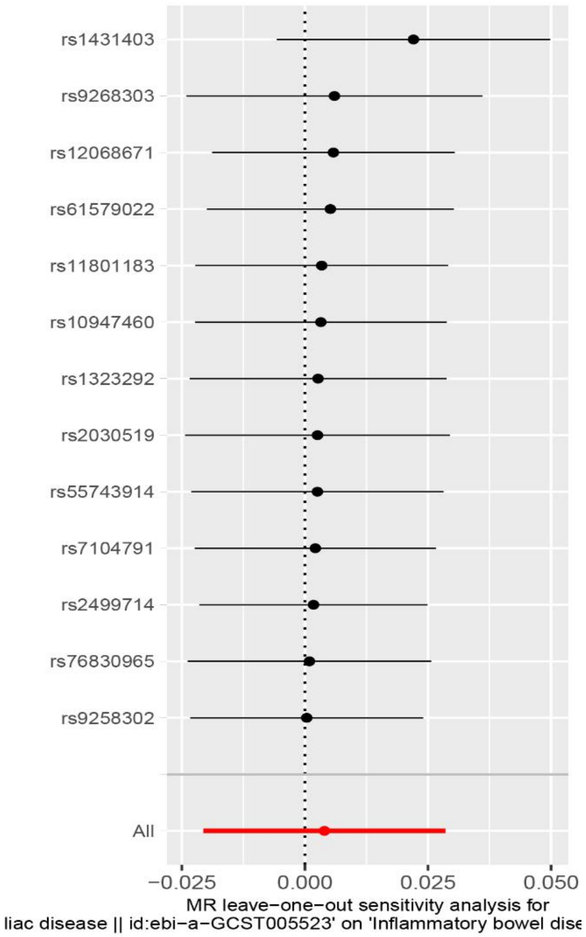

Figure S4. “Leave-one-out” sensitivity analysis of causal effects of celiac disease on inflammatory bowel disease.

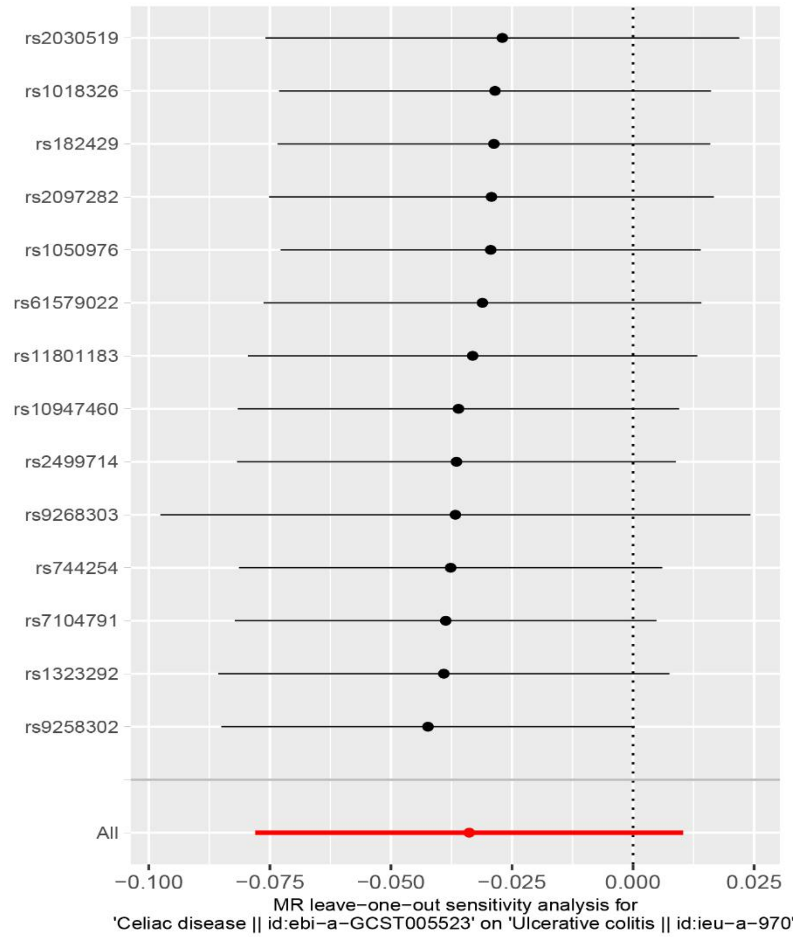

Figure S5. “Leave-one-out” sensitivity analysis of causal effects of celiac disease on ulcerative colitis.

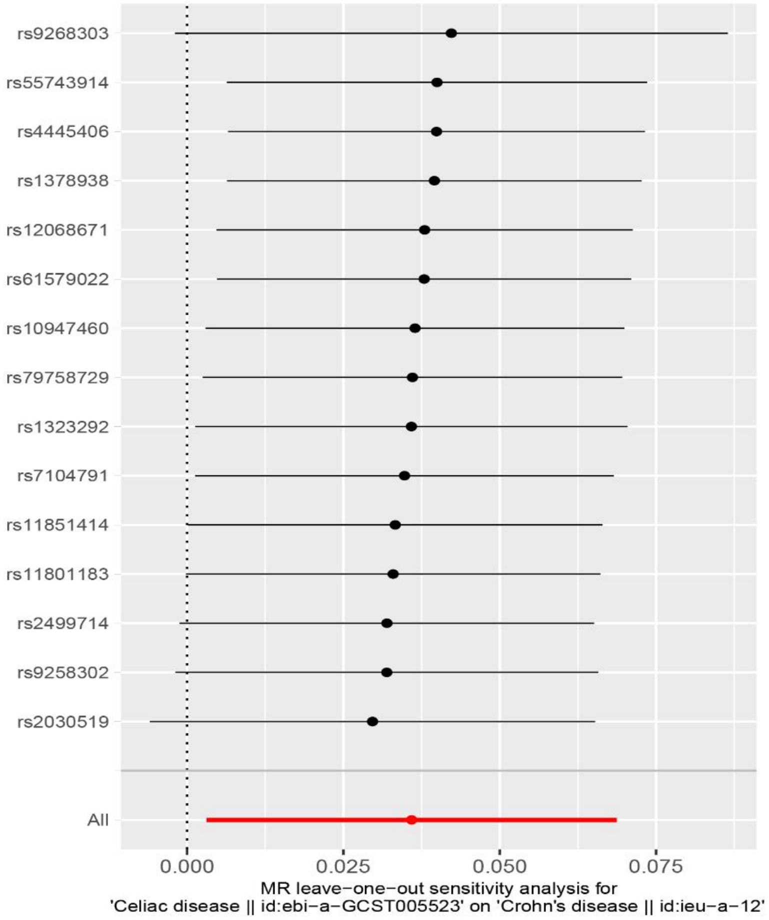

Figure S6. “Leave-one-out” sensitivity analysis of causal effects of celiac disease on Crohn’s disease.
